# AMPA receptor antagonist perampanel affects glioblastoma cell growth and glutamate release *in vitro*

**DOI:** 10.1371/journal.pone.0211644

**Published:** 2019-02-04

**Authors:** Falko Lange, Konrad Weßlau, Katrin Porath, Max Frederik Hörnschemeyer, Carina Bergner, Bernd Joachim Krause, Christina Susanne Mullins, Michael Linnebacher, Rüdiger Köhling, Timo Kirschstein

**Affiliations:** 1 Oscar-Langendorff-Institute of Physiology, Rostock University Medical Center, Rostock, Germany; 2 Center for Transdisciplinary Neurosciences Rostock, University of Rostock, Rostock, Germany; 3 Department of Nuclear Medicine, Rostock University Medical Center, Rostock, Germany; 4 Department of Surgery, Rostock University Medical Center, Rostock, Germany; Kyung Hee University, REPUBLIC OF KOREA

## Abstract

Epileptic seizures are frequent in patients with glioblastoma, and anticonvulsive treatment is often necessary. While clinical guidelines recommend all approved anticonvulsants, so far it is still unclear which of the available drugs is the best therapeutic option for treating glioma-associated seizures, also in view of possible anti-tumorigenic effects. In our study, we employed four patient-derived low-passage cell lines of glioblastoma and three cell lines of brain metastases, and challenged these cultures with four anticonvulsants with different mechanisms of action: levetiracetam, valproic acid, carbamazepine and perampanel. Cell proliferation was determined by bromodeoxyuridine incorporation. To further analyze the effects of perampanel, apoptosis induction was measured by caspase 3/7 activation. Glutamate release was quantified and glucose uptake was determined using ^18^F-fluorodeoxyglucose. Real-time polymerase chain reaction was employed to assess the expression of genes associated with glutamate release and uptake in brain tumor cells. Of the four anticonvulsants, only perampanel showed systematic inhibitory effects on cell proliferation, whereas all other anticonvulsants failed to inhibit glioma and metastasis cell growth *in vitro*. Metastasis cells were much more resistant to perampanel than glioblastoma cell lines. Glucose uptake was attenuated in all glioblastoma cells after perampanel exposure, whereas cell death via apoptosis was not induced. Extracellular glutamate levels were found to be significantly higher in glioblastoma cell lines as compared to metastasis cell lines, but could be reduced by perampanel exposure. Incubation with perampanel up-regulated glutamine synthetase expression in glioblastoma cells, whereas treatment with valproic acid and levetiracetam downregulated excitatory amino acid transporter-2 expression. Overall, our data suggest that perampanel acts as an anticonvulsive drug and additionally mediated anti-tumorigenic effects.

## Introduction

Epileptic seizures are a common feature of primary brain tumors. In low-grade gliomas, up to 90% of the patients suffer from seizures, whereas in high-grade glioma (WHO grade III–IV) patients, at least 25–60% present with tumor-associated epilepsy [1–3]. Therefore, often not only an anti-tumorigenic treatment is necessary, but there is also a need for the control of epileptic seizures. To treat seizures, a broad range of antiepileptic drugs (AED) with different mechanisms of action are available, with many of the drugs having multiple or unknown targets [4,5].

Up to now, it is still unclear which of the available anticonvulsants may represent the best therapeutic option to treat glioma-associated seizures, and which in addition may even have anti-tumorigenic effects. In first-line anticonvulsive monotherapy, often levetiracetam (LEV) and valproic acid (VPA) are considered [6,7]. VPA, which may act as an enhancer of inhibitory GABA effects by blocking of GABA-transaminase and increased GABA synthesis [8], could also show anti-tumorigenic effects by acting as a histone deacetylase inhibitor [9,10]. Whether or not VPA treatment prolongs glioma patient survival in combination with temozolomide and radiotherapy is a matter of debate [11,12]. The second drug is LEV, an AED that exerts its anticonvulsive effects via binding of the SV2A protein at synaptic vesicles, although the exact mechanism of action remains unclear. LEV has a broad therapeutic range, a rapid onset of action and due to an excellent tolerability it is often prescribed. Anti-tumorigenic effects that were achieved in cell cultures studies [13,14] could not be transferred into successful clinical trials. Thus, Kim *et al*. showed that LEV may support a longer survival in patients with glioma [15], an analysis of four clinical trials failed to show a survival benefit of LEV [11].

Although the underlying pathophysiology of glioma and tumor-associated epilepsy is not fully understood, it is generally accepted that glutamate released by tumor cells plays a major role [4,16]. High levels of extracellular glutamate in the synaptic space promote an epileptic phenotype [17,18], stimulate proliferation and invasion of tumor cells [19,20]. Therefore, AEDs targeting glutamate action are promising candidates for the treatment of glioma-associated seizures. Talampanel–an α-amino-3-hydroxy-5-methyl-4-isoxazolepropionic acid (AMPA) receptor antagonist—was tested in a phase II trial in combination with temozolomide and radiotherapy, and patients on talampanel therapy showed a longer survival than patients without AMPA receptor antagonist [21]. However, in a second phase II trial from the same year, no benefit from talampanel therapy with respect to survival was observed [22]. Another noncompetitive AMPA receptor antagonist is perampanel (PER) [23,24]. Perampanel was used in drug-resistant epilepsy of gliomas, and seizure-free periods could be achieved [25–27]. As mentioned above, it remains controversial which AED is the best option to mediate not only an anticonvulsive, but also an anti-tumoral efficacy to glioma cells. Therefore, we studied the biological and molecular effects of four clinically available anticonvulsants (LEV, VPA, PER and the sodium channel blocker carbamazepine (CBZ)), on brain tumor cells. To mimic native conditions within the original tumor closely *in vitro*, we employed patient-derived low-passage cells lines of glioblastoma and metastasis cells [28].

Of the four therapeutics, only PER was able to inhibit cell growth in three out of four glioblastoma cell lines tested, whereas metastasis cells were unaffected by low doses of perampanel. Furthermore, PER attenuated glucose uptake and extracellular glutamate levels of tumor cell cultures, but showed no effect on gene expression profile of genes associated with glutamate release/reuptake. Taken together, PER appears to exert an antitumor efficacy in addition to its AED profile.

## Materials and methods

### Cell culture

In this study, four low-passage glioblastoma (WHO grade IV) cell lines (HROG) and three low-passage brain metastasis cell lines (HROBM) were employed (Table 1). Brain metastasis cell lines were derived from metastases localized in the brain found in patients with a primary colorectal carcinoma or a non-small lung carcinoma, respectively. All cell lines used in the experiments did not exceed passage 50. Glioblastoma or metastasis tumors were obtained from surgery following informed written patient consent. All procedures were approved by the Ethics Committee of the University Medicine of Rostock (reference ID: A 2009/34) in accordance with generally accepted guidelines for the use of human material. The establishment of the glioblastoma cell lines from primary brain tumor specimen was previously described in detail in Mullins *et al*. 2013 [28]. Genetic and epigenetic markers were used to classify glioblastoma to molecular subtypes [29]. HROG02 and HROG24 were identified as the proneural subtype, HROG05 and HROG15 were classified as the mesenchymal subtype [30]. HROG02, HROG15, and HROG24 were obtained from untreated primary tumors, while the HROG05 cell line was established from a relapsed glioblastoma after previous chemoradiotherapy. All metastasis cell lines were generated in the same way. Briefly, brain tumor tissue was minced, washed in PBS and cell suspension was seeded in culture medium. Outgrowing cells were passaged 2–3 times and expand afterwards for subsequent experiments. All cell lines were cultured in Dulbecco’s Modified Eagle Medium: Nutrient Mixture F-12 (DMEM/F12; from Merck Millipore, Darmstadt, Germany) with 10% fetal calf serum (FCS; Biochrom, Berlin, Germany) at 37 °C in a 5% CO_2_ humidified atmosphere. Routinely, cell culture supernatants were tested for mycoplasma contamination employing MycoAlert Mycoplasma Detection Kit (Lonza, Basel, Switzerland).

**Table 1:** Cell lines and clinical data.

Tumor ID	Gender/Age	Tumor Location	Tumor species (origin)
HROG02	M/68	R; parietooccipital	primary glioblastoma
HROG05	F/60	L; temporal	primary glioblastoma
HROG15	M/56	R; parietal	primary glioblastoma
HROG24	F/73	L; occipital	primary glioblastoma
HROBML01	M/67	cerebrum	non-small cell lung cancer
HROBML03	F/47	cerebrum	non-small cell lung cancer
HROBMC01	F/60	cerebrum	colon carcinoma

#### Quantification of DNA synthesis

DNA synthesis was quantified employing a 5-bromo-2´-deoxyuridine (BrdU) incorporation assay kit (Roche Diagnostics, Mannheim, Germany). Therefore, the cells were seeded in 96 half-area microplates at equal seeding densities and allowed to adhere overnight in complete culture medium. On the following day, culture medium was substituted with medium supplemented with CBZ, LEV, VPA (all three from Selleck Chemicals, Houston, TX, USA) or PER (kindly gifted from Eisai Inc., Tokyo, Japan) at the indicated doses. After an incubation period of 40 h, BrdU incorporation was initiated by adding BrdU solution at a final concentration of 10 μM. Another 8 h later, labeling was stopped, and BrdU uptake was measured according to the manufacturer´s instructions employing a GloMax-Multi Detection System (Promega, Madison, WI, US).

#### Analysis of cellular DNA content by flow cytometry

Glioblastoma cells growing in 6-well plates in complete culture medium were exposed to PER for 48 h as indicated. For the detection of the cellular DNA content, trypsinized glioblastoma cells were pelleted by centrifugation, washed twice with PBS (pH 7.4) and resuspended in ice-cold 70% ethanol for at least 12 h at -20 °C. After additional washing steps, the cells were incubated for 1 h in 250 μL PBS-0.1%Tween20 supplemented with 0.1 mg/mL RNase A (Roboklon, Berlin, Germany) on ice. Subsequently, 50 μl propidium iodide (10 μg/ml, Sigma Aldrich, Deisenhofen, Germany) was added and the samples were subjected to cytofluorometric analysis. 10,000 events were measured for each sample using a FACSCalibur cytometer (BD Biosciences, Heidelberg, Germany). The cell cycle distribution was calculated using software tools CellQuest and ModFit LT. Cells of the Sub-G1 peak were considered apoptotic.

#### Caspase 3/7 activation assay

Caspase-3/7 activity was measured in a cell-based assay using the luminogenic substrate Z-DEVD-aminoluciferin with a tetrapeptide sequence specific for caspase-3/7. Therefore, the cells were seeded in 96-well plates in complete culture medium. On the next day, PER, or solvent (dimethylsulfoxide, DMSO) was added and the incubation was continued for an additional time of 6 h. Afterwards, caspase activity was measured following the instructions of the manufacturer (Promega). Briefly, the cell culture plates were removed from the incubator and the luminogenic substrate was added. Assay plates were incubated at 22 °C for 1 h before recording the luminescence with a GloMax microplate reader. For the generation of caspase 3/7-positive control samples, the cells were treated with 1 μM staurosporine (Sigma-Aldrich) for 2 h and caspase activity was determined as described above.

#### 2-Deoxy-2-[18F] fluoroglucose uptake (^18^F-FDG)

To analyze the effects of perampanel on glucose uptake, HROG cells and brain metastasis cells were seeded in 24-well plates at equal seeding densities and allowed to adhere overnight in complete culture medium. Afterwards, PER was added for 48 h. Next, complete culture medium was substituted by Dulbecco’s modified Eagle medium (DMEM; Fisher Scientific, Schwerte, Germany) without FCS, glucose, glutamine and phenol red (but supplemented with PER or solvent as before), and incubated for 1 h before ^18^F-FDG (0.5 MBq/ml culture medium) was added to each well. 30 min later, incubation was terminated by aspirating the medium and rinsing the cell layer three times with ice-cold PBS. The cells were solubilized using 0.1 mol/l NaOH, and incorporated ^18^F activity was determined using a gamma counter (WIZARD2 10-Detector Gamma Counter, PerkinElmer, Waltham, MA, US). Counts per minute were normalized on total protein level of the cells using a commercial Bradford assay (Bio-Rad Laboratories, Munich, Germany). ^18^F-FDG uptake of cells treated with perampanel was expressed as the percentage of control cells exposed to DMSO only (n = 9 per cell line and experimental condition).

#### Detection of extracellular glutamate levels

HROG cells and brain metastasis cells were seeded in 6-well plates at equal seeding densities and allowed to adhere overnight in complete culture medium. On the next day, medium was substituted by DMEM (w/o L-glutamine, w/o FCS) and perampanel or DMSO was added. Supernatants of subconfluent growing cells were collected for a total of 24 h and glutamate levels were determined by using the Glutamate Assay Kit (abcam, Cambridge, UK) following the instructions of the manufacturer. Extracellular glutamate levels were normalized to the total protein level of the sample. Extracellular glutamate levels of the cells treated with perampanel were expressed as the percentage of control cells exposed to DMSO only (n≥10 per cell line and experimental condition).

#### Quantitative reverse transcriptase-PCR

To study the expression of genes associated with glutamate release [31], cells were seeded in 12-well plates at equal seeding densities and allowed to adhere overnight in complete culture medium. On the next day, medium was substituted and solvent or anticonvulsants at two different doses were added for 48 h. Afterwards, total RNA was isolated with TRIZOL reagent (Thermo Fisher Scientific, Karlsruhe, Germany) according to the manufacturer’s instructions. Afterwards, 1 μg RNA was reverse transcribed into cDNA by M-MLV Reverse Transcriptase, random hexamer primers, dNTP mix (10 mM each) and RNasin plus ribonuclease inhibitors (all from Promega, Mannheim, Germany). Traces of genomic DNA were removed employing the DNA-free DNA removal kit (Thermo Fisher Scientific). Relative quantification of target cDNA levels by real-time PCR was done with SYBR Green qPCR Master mix (Bimake, Munich, Germany) using primers for branched chain amino acid transaminase 1 (*BCAT1)*, excitatory amino acid transporter 2 (EAAT2, *SLC1A2* gene), glutamine synthetase (*GLUL*), isocitrate dehydrogenase 1 (*IDH1*), amino acid transport system X_c_^-^ (*SCL7A11*) and glyceraldehyde 3-phosphate dehydrogenase (*GAPDH*, house-keeping gene control; Table 2). PCR conditions were: 95 °C for 2 min; followed by 40 cycles of 15 s at 95 °C/ 15 s at 55 °C/ 20 s at 68 °C. For each independent sample (n = 5–6 per cell line and experimental condition), the relative expression of mRNA compared to GAPDH was calculated according to the equation ΔCt = Ct_*target*_ − Ct_*GAPDH*_. The relative amount of target mRNA was expressed as ΔCt or 2^-ΔCt^.

**Table 2:** Primers used for reverse transcriptase PCR.

Gene	Forward Primer	Reverse Primer
*BCAT1*	5’-CAA CTA TGG AGA ATG GTC CTA AGCT-3’	5’-TGT CCA GTC GCT CTC TTC TCT TC-3’
*SLC1A2 (EAAT2)*	5’-CCT CTT AAG CCC CGT CAA GG-3’	5’-CCT CTT AAG CCC CGT CAA GG-3’
*GLUL*	5’-TCA TCT TGC ATC GTG TGT GTG-3’	5’-CTT CAG ACC ATT CTC CTC CGG-3’
*IDH1*	5’-CGG TCT TCA GAG AAG CCA TT-3’	5’-GCA AAA TCA CAT TAT TGC CAA C-3’
*SCL7A11(xCT)*	5’-TGC TGG GCT GAT TTA TCT TCG-3’	5’-GAA AGG GCA ACC ATG AAG AGG-3’
*GAPDH*	5’-CCA CTC CTC CAC CTT TGA C-3’	5’-ACC CTG TTG CTG TAG CCA-3’

#### Statistical analysis

Values were expressed as mean ± standard error of the mean (SEM) or were presented as single data values (qPCR data only) for the indicated number of samples (n) per experimental protocol. Statistical analysis was performed with SigmaPlot 13.0. Mean group differences were checked using nonparametric analysis of variance employing the Kruskal-Wallis test before for multiple comparisons subgroups were tested with post hoc Dunn’s test. Comparisons of two independent groups were performed with Mann-Whitney U test. For the analysis of extracellular glutamate levels, a two-way ANOVA followed by Bonferroni t-test was used. A significance level of p < 0.05 was considered to be statistically significant (indicated by asterisks).

## Results

### Perampanel, but not other anticonvulsants exhibit anti-proliferative effects on glioblastoma cell lines

**Fig 1:**
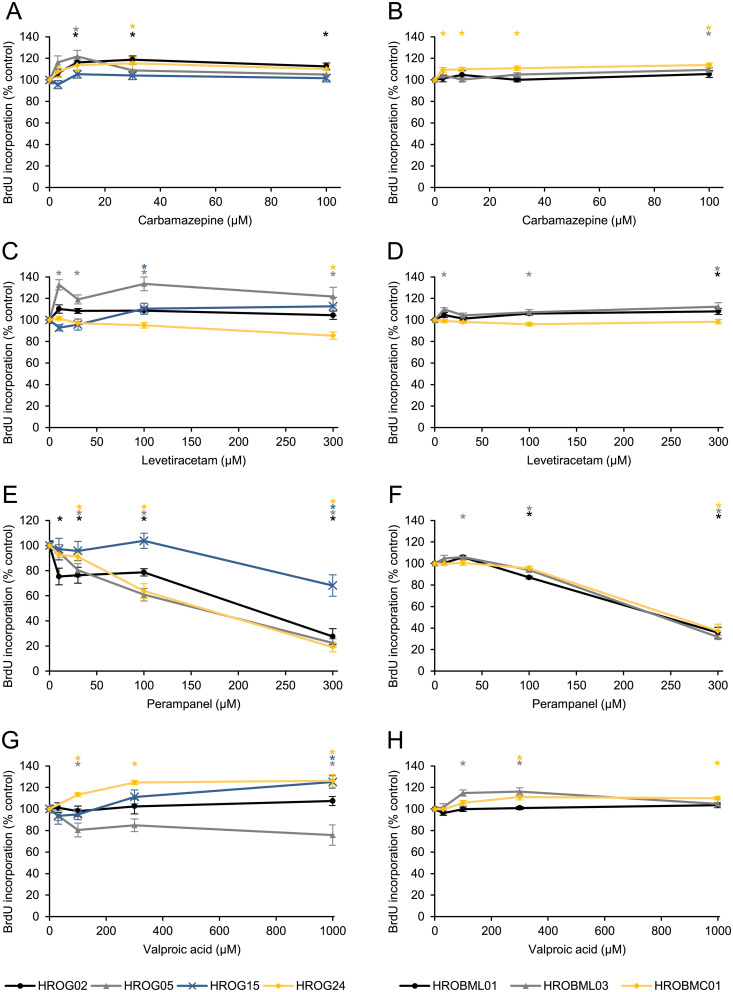
Effects of anticonvulsants on DNA synthesis of glioblastoma and brain metastasis low passage cell lines. Glioblastoma cells (HROG02, HROG05, HROG15, HROG24) and brain metastasis cells (HROBL01, HROBML03 and HROBMC01) growing in 96-well half-area microplates were treated with (A, B) carbamazepine, (C, D) levetiracetam, (E, F) perampanel or (G, H) valproic acid as indicated for 48 h, before DNA synthesis was assessed with the BrdU incorporation assay. One hundred percent BrdU incorporation corresponds to cells cultured without anticonvulsant. Data are presented as mean ± SEM (n ≥ 12 separate cultures); *p<0.05 versus control cultures (Kruskal-Wallis test with post hoc Dunn’s test).

This study aims to analyze the effects of clinically used anticonvulsants on glioblastoma and brain metastasis cells. Therefore, four low-passage glioblastoma and three metastasis cell lines derived from primary or secondary tumors were chosen. In initial experiments, the tumor cell lines were exposed to different doses of CBZ, LEV, PER or VPA, respectively, and cell proliferation was assessed by measuring the incorporation of BrdU into newly synthesized DNA (Fig 1). Anticonvulsant doses range from therapeutic to supra-therapeutic levels in patients’ sera as reported elsewhere [5,32].

Carbamazepine showed no inhibitory effects on the cell proliferation, but slightly increased DNA synthesis in 3/4 glioblastoma and 2/3 metastasis cell lines (see colored asterisks in Fig 1A and 1B, respectively).

The response pattern of LEV was similar to CBZ treatment with no effect on cell proliferation or even an enhanced DNA synthesis after incubation with the anticonvulsant (Fig 1C and 1D). Nevertheless, at a supra-therapeutic concentration (plasma level ranges between 18–180 μM) [5], an inhibitory effect (86%±3%) could be observed in HROG24 cells (Fig 1C, yellow symbol).

In contrast, anti-proliferative effects of PER were detected in all glioblastoma cell lines (Fig 1E), and–even more important–three out of four cell lines showed a response at rather low concentrations (10–30 μM). HROG02 cells were most sensitive to PER with a significantly reduced proliferation at the low concentration of 10 μM (75%±7%), while PER less potently inhibited the proliferation of HROG05 and HROG24 cells (80%±5% and 91%±3%, respectively, 30 μM PER). Only the HROG15 cell line was resistant to the drug up to 300 μM PER (Fig 1E, blue symbols). In contrast to CBZ and LEV, we never found an increased DNA synthesis following PER incubation. On the other hand, human brain metastasis cells presented a less sensitive response pattern (Fig 1F). Only at high concentrations (300 μM), PER showed some inhibitory effect on proliferation. The histone deacetylase blocker VPA inhibited proliferation of HROG05 cells at therapeutic concentrations (81%±6% at 100 μM), but other cells lines showed unchanged or even significantly enhanced levels of BrdU incorporation (Fig 1G and 1H).

Taken together, treatment with PER exhibited medium to strong anti-proliferative effects on glioblastoma cell lines, but CBZ, VPA and LEV failed to decrease proliferation.

### Perampanel mediated antiproliferative effects are not due to enhanced apoptosis

**Fig 2:**
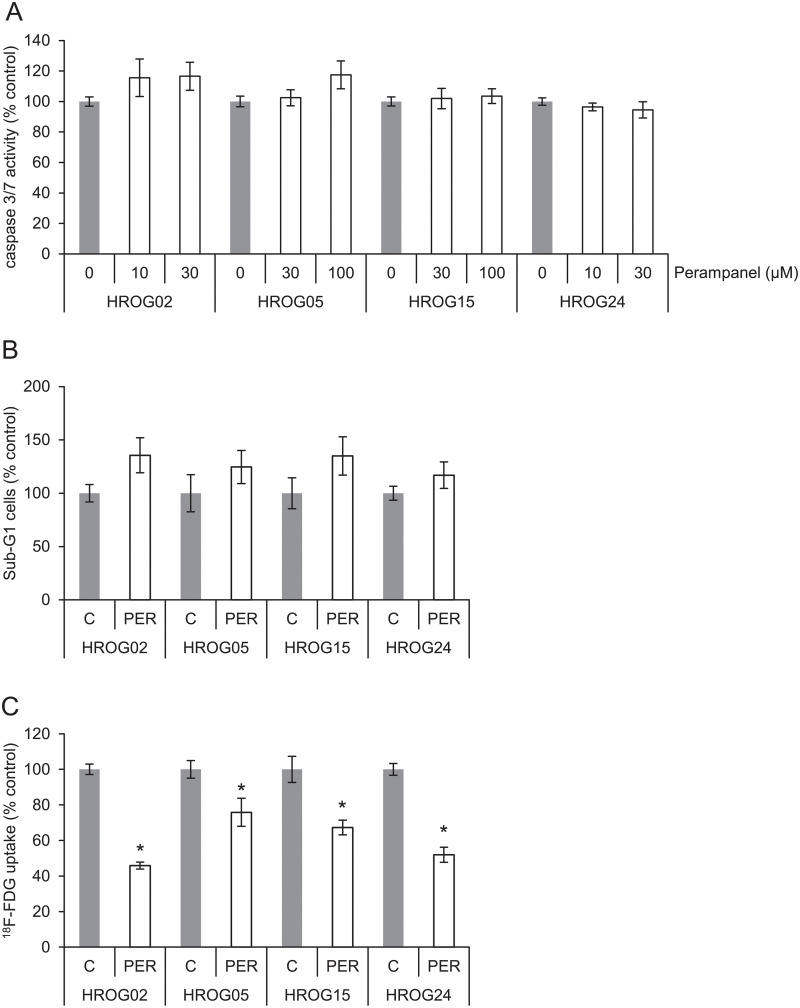
Cell death, caspase activation, and FDG uptake. (A) Subconfluent-growing glioblastoma cells were challenged with PER or solvent control DMSO, and caspase 3/7 enzyme activity was determined employing a colorimetric kit. Data are presented as mean ± SEM (n = 7–8 separate cultures). No significant caspase activation was observed (Kruskal-Wallis test with post hoc Dunn’s test). (B) Glioblastoma cells were exposed to highest dose of PER from caspase activation analysis for 48 h and of apoptotic (Sub-G1 peak) cells were estimated by cytofluorometric quantification. The portion of dead/apoptotic cells is expressed as percent of solvent treated control cultures. Data from *n* ≥ 5 separate cultures were used to calculate mean values ± SEM. No significant change in Sub-G1 fraction was observed (Mann-Whitney U test). (C) Glioblastoma cells were labelled with ^18^F-FDG, and tracer uptake was quantified. Counts per minute were normalized to the protein content of the samples. One hundred percent ^18^F-FDG uptake corresponds to solvent-treated tumor cells (n = 9; mean values ± SEM); *p<0.05 versus control cultures (Mann-Whitney U test).

Since PER showed anti-proliferative effects on the glioblastoma cells, we asked the question whether cell death by apoptosis could explain these effects. As a surrogate marker for apoptosis, activation of caspase 3 and 7 was determined. At concentrations of 10–100 μM (Fig 2A), the AMPA receptor antagonist had no effect on the activation of caspase 3/7. In contrast, staurosporine (1 μM, 2 h)-treated cell cultures showed increased caspase activation and were used as positive controls for apoptosis induction (HROG02: 227%±29%, HROG05: 406%±22%, HROG15: 871%±106%, HROG24: 273%±24%). Additionally, a cell cycle analysis was performed. The cells were challenged with the highest dose of PER used in caspase activation experiments (30–100 μM). No changes in the apoptotic/dead fraction of the cells were detected (Fig 2B). These results indicate that enhanced apoptosis was unlikely to be involved in the anti-proliferative effects of PER. Remarkably, no changes in cell cycle distribution at all were detected (S1 Fig).

### Perampanel attenuates glucose uptake in glioblastoma cells

Next, we analyzed PER effects on cell metabolism. Therefore, 2-deoxy-2-(18F)fluoro-D-glucose (^18^F-FDG) uptake was chosen as a surrogate marker, and the cells were challenged with 30 μM PER (Fig 2C). When normalized to solvent-incubated cells, PER displayed a significantly inhibitory effect on glucose uptake on all cell lines (Fig 2C). Thus, the anti-proliferative action of PER may be partly due to a compromised cell metabolism in glioblastoma cells as evidenced by reduced ^18^F-FDG uptake.

### Perampanel may lower extracellular glutamate levels of glioblastoma and brain metastasis cell cultures

**Fig 3:**
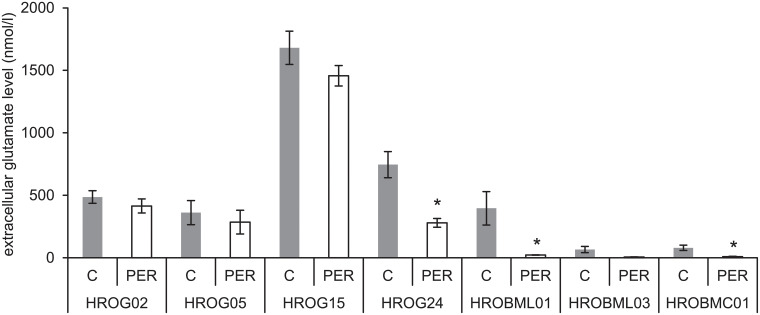
Glutamate release of glioblastoma and brain metastasis cells. In subconfluent cell cultures, supernatants (w/o FCS) were collected for a total of 24 hours (± PER) and glutamate levels were determined. Extracellular glutamate levels were normalized to total protein levels of the cells. Data are presented as mean ± SEM (n≥14), *p<0.05 vs. solvent control (Mann-Whitney U test). Multiple comparisons versus control groups (two-way ANOVA with Bonferroni t-test) demonstrated an overall higher glutamate level in the supernatant of glioblastoma cells than in the supernatant of metastasis cells (p<0.001). Additionally, the two-way ANOVA also revealed a significant treatment effect, i.e. PER attenuated extracellular glutamate levels across all cell cohorts (p = 0.046).

Glutamate is the major excitatory neurotransmitter in the human brain and glutamate levels in the cerebral extracellular fluid were found to be elevated in patients with glioma [33,34]. Since PER acts as an antagonist of AMPA receptors and glutamate is believed to be trophically important for glioma cells [7], we measured the extracellular glutamate levels of glioblastoma and metastasis cell cultures. The results indicate that an incubation with PER significantly reduced the extracellular glutamate levels in HROG24 as well as in the metastasis cell lines HROBML01 and HROBMC01 (Fig 3). Additionally, a two-way ANOVA (factor cell culture, i.e. glioblastoma versus metastasis and factor treatment, i.e. PER versus control media) with Bonferroni posthoc test revealed that glioblastoma cell cultures on the one hand accumulate significantly higher extracellular glutamate levels than metastasis cell cultures on the other hand (p<0.001). Furthermore, PER-treated cultures contained significantly less extracellular glutamate levels than solvent-treated tumor cell cultures (p = 0.046; two-way ANOVA followed by Bonferroni t-test).

### Transcriptional effects of anticonvulsants

**Fig 4:**
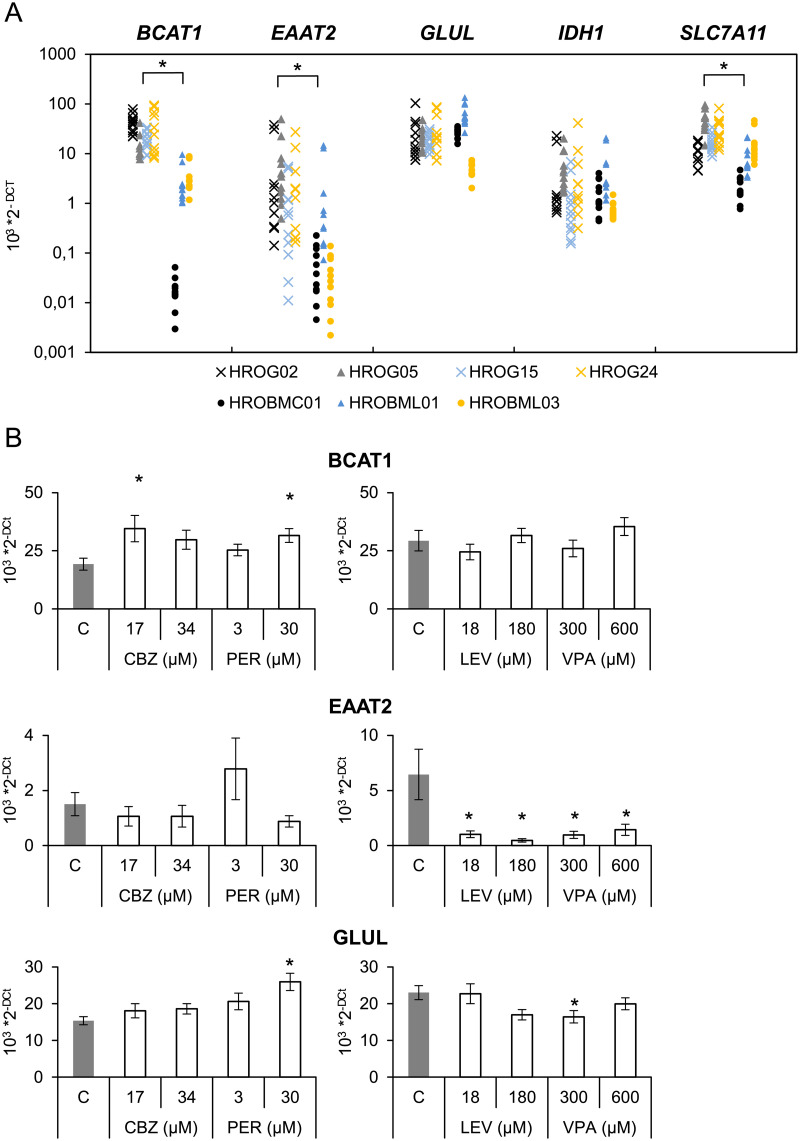
Effects of anticonvulsants on gene expression. Both, glioblastoma and metastasis cells were seeded in 12-well plates. On the next day, medium was exchanged and the cells were exposed to carbamazepine, levetiracetam, perampanel or valproic acid at the indicated doses for 48 h. Subsequently, the mRNA expression of the indicated genes and house-keeping control *GAPDH* was analyzed by real-time PCR. (A) Relative amounts (2^-ΔCt^) of target mRNA of control cultures were compared for both cell entities (n ≥ 10 per cell line), *p<0.05 versus glioblastoma cells (Mann-Whitney U test). (B) Furthermore, effects of anticonvulsants were analyzed. Only genes with significant changes are shown in Fig 4B (n = 5–6 separate cultures per cell line; *p<0.05 versus control cultures (Kruskal-Wallis test with post hoc Dunn’s test)).

In order to obtain more detailed information on the molecular effects of all four anticonvulsants on gene expression of a panel of key proteins responsible for glutamate metabolism and release [7,35], a real-time PCR analysis was employed. We chose anticonvulsant concentrations ranging from therapeutic to supra-therapeutic human serum levels [5,32]. First, we compared glioblastoma and metastasis cells, and we could confirm that metastasis cell cultures expressed significantly less *BCAT1*, *EAAT2* and *SLC7A11* as compared to glioblastoma cells suggesting that these genes may represent a glioblastoma-typical gene profile (Fig 4A).

Following 48 h of incubation with two different doses of anticonvulsants, significant effects could only be found in the expression of *BCAT1*, *EAAT2* and *GLUL* in glioblastoma cells (Fig 4B), whereas no changes in *IDH1* and *SLC7A11* expression were found (S2 Fig). Expression of *BCAT1* was significantly elevated by CBZ and PER, whereas *GLUL* expression was significantly increased only by the AMPA receptor inhibitor PER. In addition, *EAAT2* expression was almost abolished by both LEV and VPA at the indicated doses. Notably, in metastasis cell cultures no transcriptional impact on gene expression was detected at all (S3 Fig).

## Discussion

Adequate control of epileptic seizures in patients with gliomas contributes significantly to the quality of life, particularly in palliative conditions when tumor remission may no longer be achieved. Currently, it is still unclear which of the available AEDs approved for the treatment of brain-tumor associated seizures could be the most beneficial drug with respect to both an anti-convulsive and anti-tumorigenic therapy. Undoubtedly, identification of an AED that beyond seizure control may also attenuate tumor progression would be of great clinical interest. To address this question, we took advantage of patient-derived low-passage cell lines of glioblastoma and brain metastasis samples. As shown for brain tumor and other cancer diseases, those *in vitro* models present a feasible tool close to the *in vivo* conditions to study drug effects in preclinical and translational research [36–38].

One major finding of our study was that among the tested AEDs with different mechanisms of action–LEV, CBZ, VPA, and PER–only the AMPA receptor antagonist PER showed systematic inhibitory effects on cell proliferation. In addition, as shown by cell cycle analysis and caspase activity assay, we found that PER did not lead to apoptosis induction which is in line with a previous study [39], but was associated with reduced cell metabolism. An inhibition of AMPA receptors led to an attenuated p44/42 MAPK or PI3K/AKT activation and reduced cell proliferation, apoptosis induction, and migration of various cell types including GBM specimens [20,40–42]. However, it is likely that further mechanisms may contribute to the anti-proliferative PER effects that have to be examined.

In marked contrast to PER, the sodium channel blocker CBZ had no effects or even promoted cell proliferation. In a recent report employing human U87MG and T98 cell lines, a constant growth inhibition of roughly 20% independent of the used CBZ concentration was observed [43], indicating that CBZ does not show concentration-dependent pharmacological effects on proliferation. In the case of LEV, we saw a similar result as with CBZ, but at supra-therapeutic levels an inhibitory effect could be detected. Intriguingly, LEV may sensitize glioma cells to temozolomide and therefore mediate anti-tumorigenic effect *in vitro* [13,14]. VPA was also described as an anticonvulsant that may lower resistance to radiation [44,45]. In our study, only HROG05 was susceptible to VPA treatment at therapeutically relevant doses with respect to BrdU incorporation. All other cell lines including metastasis cultures were VPA-resistant or cell growth was even increased. In line with our observation, Eckert *et al*. 2017 showed that VPA had only small anti-proliferative effects on human GBM cell lines, but the authors were unable to detect an impairment of survival or a lower radioresistance of temozolomide-treated primary spheroid cultures [46]. When used in dose escalation experiments that excess patient plasma levels [5], one may find inhibitory effects by VPA [47–50].

Glucose is one of the major metabolic substrates of cancer cells, as it is a primary source for energy generation, nucleotide as well as amino acid synthesis [51,52]. We used ^18^F-labeled glucose as a surrogate marker to estimate metabolic effects of PER. Intriguingly, all four tested GBM cell lines–even PER-resistant HROG15—showed a significantly reduced uptake of glucose. Cells that proliferate have a high demand for glucose. Therefore, an attenuated glucose uptake is in line with the anti-proliferative effects of PER, but cells could also be primed for apoptosis as a function of p53 expression [53]. However, glucose is not the only relevant metabolic substrate. As shown by Mashimo *et al*. 2014, acetate is also an important bioenergetical substrate for gliomas that could in part compensate for the glucose-dependence [54].

To our best knowledge, levels of glutamate released by colon or lung cancer metastasis cells within the brain were not determined to far. Brain metastases grow less infiltrative and provoke less neuronal excitability [5]. We found overall reduced glutamate levels in metastasis cell cultures as compared to GBM cells. This finding correlates with a lower expression of *BCAT1*, *EAAT2*, and *SLC7A11* in metastasis cells, and also indicates a less-distinct glutamatergic phenotype of the metastasis cells [16]. SLC7A11 (xCT) is a glutamate/cystine antiporter highly expressed in glioma cells, where it was identified as a key-driver in glutamate release [18,55–57]. Recently, in two studies xCT was identified as a biomarker for seizures underlining its importance in glioma-associated epilepsy [58,59]. Branched-chain amino acid transaminase 1 (BCAT1), an enzyme that initiates the catabolism of branched-chain amino acids and therefore raises the level of intracellular glutamate, promotes glioma proliferation *in vitro* and *in vivo* [35]. In turn, inhibition of BCAT1 led to a lower amount of glutamate excretion of glioma cells [35]. Furthermore, reuptake of glutamate into glioma cells is impaired because expression of EAAT2, a sodium-dependent glutamate transporter, is often downregulated in gliomas [18,57,60]. A lower EAAT2 expression in metastasis cells could possibly reflect the different origin of the tumor cells. One key function of astrocytes is neurotransmitter homeostasis including rapid removal of glutamate from the synaptic space [61], and both enhanced xCT and BCAT1 expression and reduced EAAT2 expression occur in gliomas [18,57–60]. Hence, we assume that both glioma cells release excessive glutamate and the uptake is too low by these cells. Both mechanisms, therefore, are presumable risk factors for seizure generation.

Another finding of our study was that PER affected the total level of glutamate in media across of all cultures. A reduction of glutamate should have neuroprotective effects to surrounding cells and also attenuate pro-tumorigenic autocrine loops that promote proliferation, invasion and migration of glioma cells (summarized in [7]). The mechanism by which PER reduces the glutamate content remains to be clarified. Since glutamate is released through SLC7A11 transporters, we initially hypothesized that PER might down-regulate SLC7A11 transcription. However, this was not the case, and glutamate release might be mediated by both SLC7A11-dependent and -independent mechanisms. With respect to our cell cycle analysis, we can exclude that the cells were arrested in a particular cycle phase following PER.

Furthermore, we asked if PER effects on glutamate release could be correlated with the expression of genes whose protein products are highly involved in glutamatergic signaling of glioma. Additionally, we also included all initially used AEDs (CBZ, LEV, and VPA). In the present study, both LEV and VPA, but not PER further reduced EAAT2 transcription which might be a rather detrimental effect of these compounds, since sodium-dependent reuptake of glutamate via EAAT2 is a principal mechanism to eliminate the neurotransmitter from extracellular space. In contrast, PER significantly enhanced GLUL gene expression. This enzyme creates glutamine from glutamate and thus lowers cytosolic glutamate levels. At least from a theoretical point of view, this enzyme thus may help decreasing extracellular glutamate levels, which could be a beneficial effect of PER in treating glioma-associated seizures. Therefore, it is an intriguing finding that anticonvulsants may change the transcription of these genes. This should be considered in future clinical decision-making when treating glioma-associated seizures.

On the other hand, gene expression analysis in brain metastasis cells did not reveal any alterations by anticonvulsive treatment. This is consistent with the finding that these compounds had either no or only weak effects on the proliferation, and is in marked contrast to the data obtained from glioblastoma cell lines presenting with an altered gene expression in response to anticonvulsive treatment, but we do not believe that different modes of action might account for this difference. Rather, it is likely to be due to the intrinsic genotype and/or phenotype of the cells. Nevertheless, we cannot exclude that so far unknown molecular targets of the drugs differ between cell lines.

In conclusion, the present study showed that PER has anti-proliferative effects on glioblastoma cell lines associated with reduced glucose uptake. Extracellular glutamate levels are generally higher in glioblastoma cell lines as compared to metastasis cell lines, but may be reduced by PER incubation. Lastly, anticonvulsants may change transcription of genes, and in this respect, PER presents with a more beneficial profile than LEV or VPA. Based on our data, we therefore suggest follow-up studies in animal models of glioma not only to test perampanel action on brain-tumor-derived seizures, but also use this compound as an add-on anticonvulsant in a radiochemotherapy regime to further evaluate its anti-tumorigenic effect under *in vivo* conditions.

## Supporting information

S1 Fig Glioblastoma cells growing in 6-well plates in complete culture medium were exposed to PER for 48 h at the indicated doses. Processing of the cells after cultivation is described in details in the Materials and Methods sections of the manuscript. (A) A cytofluorometric analysis was performed employing a FACSCalibur cytometer. The cell cycle distribution was calculated using software tools CellQuest and ModFit LT (HROG02 plots are shown as example). (B) Data from *n* ≥ 5 separate samples were used to calculate mean values ± SEM. No significant changes were determined by employing a Mann-Whitney U-test (control vs. PER-treated cultures).
(PDF)

S2 Fig Glioblastoma cells were seeded in 12-well plates. On the next day, medium was exchanged and the cells were exposed to carbamazepine, levetiracetam, perampanel or valproic acid at the indicated doses for 48 h. Subsequently, the mRNA expression of the indicated genes and house-keeping control GAPDH was analyzed by real-time PCR. Relative amounts (2^-ΔCt^) of target mRNA of control cultures were compared. No significant changes were determined by employing a Kruskal-Wallis test with post hoc Dunn’s test.
(PDF)

S3 Fig Metastasis cells were seeded in 12-well plates. On the next day, medium was exchanged and the cells were exposed to carbamazepine, levetiracetam, perampanel or valproic acid at the indicated doses for 48 h. Subsequently, the mRNA expression of the indicated genes and house-keeping control GAPDH was analyzed by real-time PCR. Relative amounts (2^-ΔCt^) of target mRNA of control cultures were compared. No significant changes were determined by employing a Kruskal-Wallis test with post hoc Dunn’s test.
(PDF)

## Abbreviations

AED: antiepileptic drug
CBZ: carbamazepine
LEV: levetiracetam
PER: perampanel
VPA: valproic acid
