# Supplementary material for: AMPA receptor antagonist perampanel affects glioblastoma cell growth and glutamate release in vitro
Source: PLoS One. 2019 Feb 4;14(2):e0211644. doi: 10.1371/journal.pone.0211644 (PMC6361447; doi:10.1371/journal.pone.0211644)
Supplement: S1 Fig — Processing of the cells after cultivation is described in details in the Materials and Methods sections of the manuscript. (A) A cytofluorometric analysis was performed employing a FACSCalibur cytometer. The cell cycle distribution was calculated using software tools CellQuest and ModFit LT (HROG02 plots are shown as example). (B) Data from n ≥ 5 separate samples were used to calculate mean values ± SEM. No significant changes were determined by employing a Mann-Whitney U-test (control vs. PER-treated cultures). (PDF) [file pone.0211644.s001.pdf]

Supporting Information

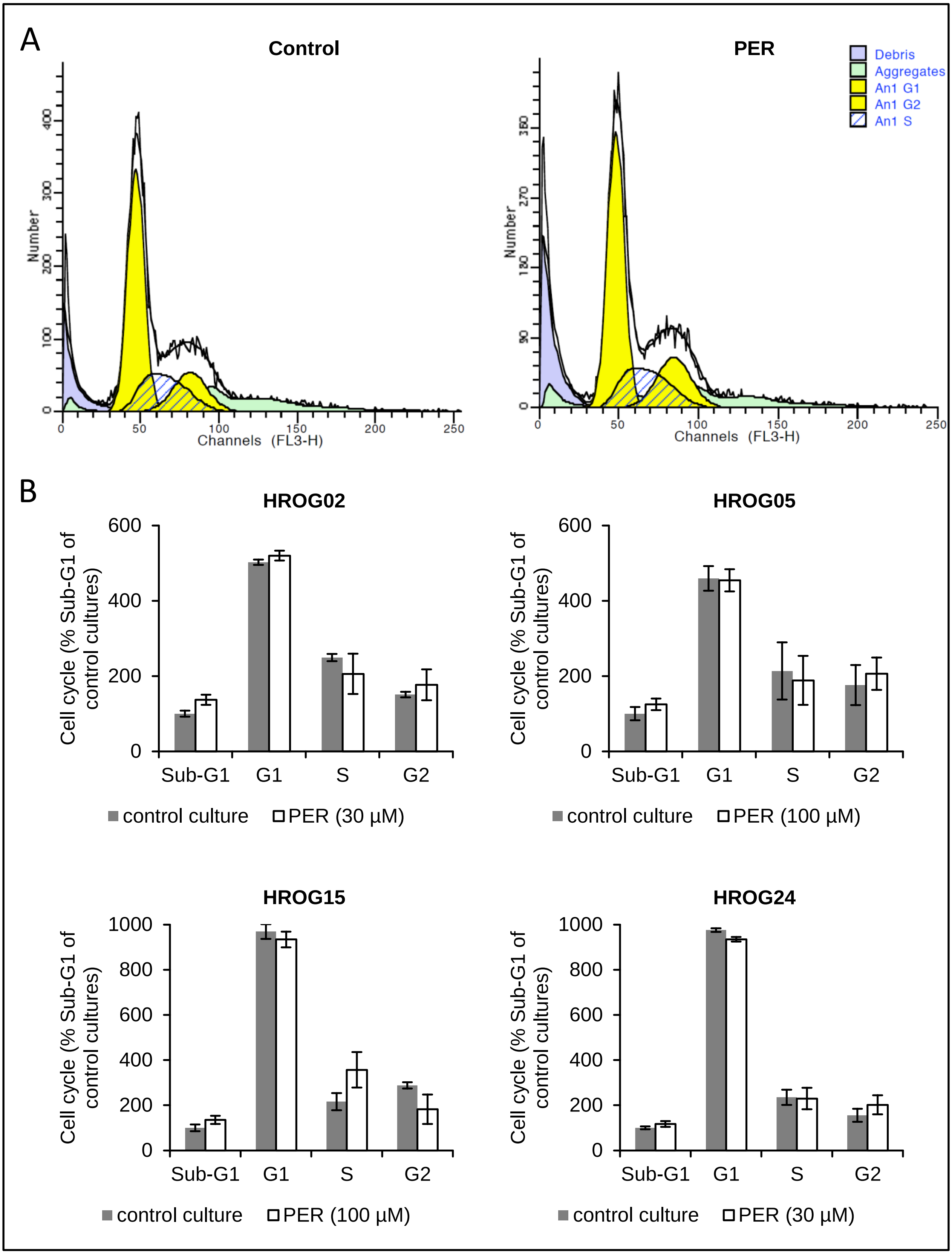

Supplementary Figure 1: Glioblastoma cells growing in 6-well plates in complete culture medium were exposed to PER for 48 h at the indicated doses. Processing of the cells after cultivation is described in details in the Materials and Methods sections of the manuscript. **A** A cytofluorometric analysis was performed employing a FACSCalibur cytometer. The cell cycle distribution was calculated using software tools CellQuest and ModFit LT (HROG02 plots are shown as example). **B** Data from $n \geq 5$ separate samples were used to calculate mean values ± SEM. No significant changes were determined by employing a Mann-Whitney U-test (control vs. PER-treated cultures).
