# Supplementary material for: AMPA receptor antagonist perampanel affects glioblastoma cell growth and glutamate release in vitro
Source: PLoS One. 2019 Feb 4;14(2):e0211644. doi: 10.1371/journal.pone.0211644 (PMC6361447; doi:10.1371/journal.pone.0211644)
Supplement: S3 Fig — On the next day, medium was exchanged and the cells were exposed to carbamazepine, levetiracetam, perampanel or valproic acid at the indicated doses for 48 h. Subsequently, the mRNA expression of the indicated genes and house-keeping control GAPDH was analyzed by real-time PCR. Relative amounts (2-ΔCt) of target mRNA of control cultures were compared. No significant changes were determined by employing a Kruskal-Wallis test with post hoc Dunn’s test. (PDF) [file pone.0211644.s003.pdf]

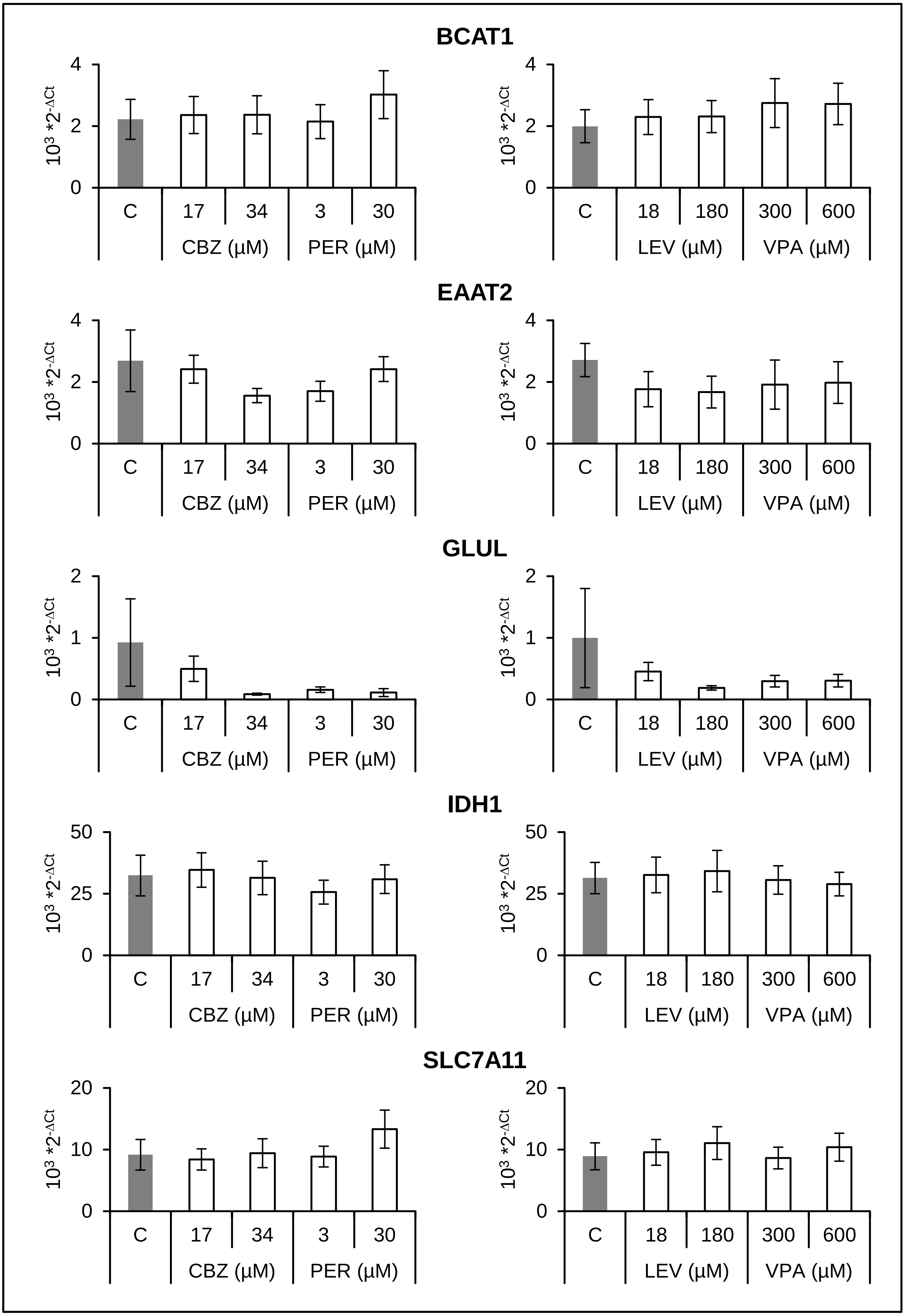

Supplementary Figure 3: Metastasis cells were seeded in 12-well plates. On the next day, medium was exchanged and the cells were exposed to carbamazepine, levetiracetam, perampanel or valproic acid at the indicated doses for 48 h. Subsequently, the mRNA expression of the indicated genes and house-keeping control GAPDH was analyzed by real-time PCR. Relative amounts ($2^{-\Delta Ct}$) of target mRNA of control cultures were compared. No significant changes were determined by employing a Kruskal-Wallis test with post hoc Dunn's test.
